# Heavy Metal(loid) Pollution Characteristics and Risk Assessment in the Water–Soil–Vegetable System of a Watershed in Southwest China

**DOI:** 10.3390/toxics14060539

**Published:** 2026-06-22

**Authors:** Mengying Li, Jinjie Zhao, Wenjing Shen, Duanyang Yuan, Chengchen Wang, Ping Xiang

**Affiliations:** 1 Open Education College, Yunnan Open University, Kunming 650023, China; dreamy1024lee@163.com (M.L.);; 2 Institute of Environmental Remediation and Human Health, School of Ecology and Environment, Southwest Forestry University, Kunming 650224, China

**Keywords:** mining-affected watershed, potentially toxic elements, potential ecological risk, health risk

## Abstract

Heavy metal(loid) pollution in watersheds surrounding mining areas originates from multiple and complex sources, posing persistent threats to terrestrial–aquatic ecosystems and human dietary safety. This study systematically investigated the pollution characteristics, spatial distribution, ecological risks and human health hazards of seven typical heavy metal(loid)s (As, Pb, Cr, Cd, Cu, Zn, and Ni) in the integrated water–soil–vegetable continuum of a mining-affected watershed in Southwest China. Field sampling was carried out in three functional zones with different mining disturbance intensities, and inductively coupled plasma mass spectrometry (ICP-MS) was used to detect heavy metal(loid) concentrations in all samples. Multiple pollution evaluation indices and the USEPA human health risk assessment model were adopted for comprehensive quantitative analysis. The results showed that 44.0% of surface water samples exceeded national permissible limits, with high-pollution areas concentrated in intensive mining zones, presenting moderate overall aquatic heavy metal(loid) pollution. Although the average concentrations of seven heavy metal(loid)s in riparian soils complied with Chinese agricultural soil screening standards, localized significant enrichment was observed for As (1.98 times), Cd (4.62 times), Cu (1.81 times), and Zn (2.72 times) compared with regional background values, causing mild comprehensive soil pollution. Farmland soils exhibited prominent Cu and Zn accumulation, and leafy vegetables in the study area suffered severe Pb and Cd pollution, with potential dietary exposure risks. Health risk assessment indicated that children face higher non-carcinogenic and carcinogenic risks than adults via soil hand-to-mouth exposure; dietary intake of vegetables leads to moderate carcinogenic risks for children caused by As and Ni exposure. Overall, this study clarifies the migration and enrichment rules of heavy metal(loid)s in the water–soil–vegetable system of mining watersheds, confirms the prominent ecological and human health risks of Cd, As and Pb in the study area, and provides targeted basic data for regional heavy metal(loid) pollution prevention and food safety management.

## 1. Introduction

Aquatic heavy metal(loid) contamination has emerged as a globally concerning environmental issue, with adverse ecological and health effects predominantly derived from intensive anthropogenic activities including mining, industrial production and agricultural cultivation [1]. For instance, toxic pollutants from domestic and industrial activities are often discharged into aquatic environments without pre-treatment; even after treatment, pollutant levels may still exceed relevant quality standards [2]. Additionally, the aquatic environment is threatened by various chemical pollutants, including fertilizers, pesticides, plastics, and heavy metal(loid)s [3]. Heavy metal(loid) contamination originates from diverse anthropogenic activities, such as mining, agricultural practices, industrial and metal(loid) operations, fossil fuel combustion, and other waste disposal methods [4]. Mining and processing of mineral resources, followed by their applications in industrial and agricultural sectors, have increased the abundance of heavy metal(loid)s in biogeochemical cycles [5]. In Yunnan Province, China, ore extraction and metal(loid) activities are the primary sources of heavy metal(loid)s in surface water systems [6].

The riparian zone acts as a critical water–land ecotone with irreplaceable ecosystem functions including nutrient retention, soil erosion control, water purification, and hydrological regulation [7]. As a transitional interface between aquatic and terrestrial environments, riparian soils serve as important sinks and secondary sources of heavy metal(loid)s. Metal(loid)s accumulated in riparian soils can re-enter surface runoff through scouring, leaching, and sediment resuspension, causing persistent watershed contamination. Nevertheless, most existing studies focus solely on a single medium (water or soil) rather than the coupled water–soil–vegetable system in mining-disturbed riparian zones, leading to unclear migration pathways and risk cascading effects. Currently, multiple methods are available for ecological risk evaluation, including the geoaccumulation index (*I_geo_*), potential ecological risk index (*RI*), standard comparison method, and bioconcentration factor; meanwhile, the hazard index (*HI*) is widely adopted for human health risk evaluation [8,9]. Establishing health risk assessment parameters is fundamental for investigating the oncogenic and non-oncogenic hazards of toxic metal(loid)s.

Heavy metal(loid) accumulation in agricultural soils severely threatens crop yield, food chain safety, and public health, owing to their non-degradability, long-term persistence, and bioaccumulation characteristics [10]. Soil pollution has become a prominent environmental challenge across China. Shifaw reported that southern China suffers more severe soil contamination than northern and western regions, with over half of provinces facing moderate to heavy pollution [11]. Heavy metal(loid)s accumulate along food chains and induce toxic effects on organisms at all trophic levels [12]. Excessive metal(loid) contents inhibit vegetable growth and reduce edible quality.

Food safety is a national priority in China, with strict supervision on heavy metal(loid) residues in agricultural products. He et al. collected 169 paired samples of rice grains and corresponding soils to conduct long-term monitoring of heavy metal(loid) concentrations in the soil–paddy–grain system in a typical electronic waste processing area (the results showed moderate soil contamination with Cd, Cu, Zn, and Ni, and approximately 20.7% of the analyzed grains exceeded the Cd regulatory limits) [13]. Multiple studies have also focused on sites such as Gannan Tungsten Co., Ltd. (Southern Jiangxi Province, China), Zhuzhou Smelter (Hunan Province, China), a lead–zinc smelter in Yunnan Province (China), and Tonglushan Copper Mine in Hubei Province (China) [14,15,16,17]. These investigations have revealed that, in most cases, metal(loid) contaminant levels within vegetables surpassed regulatory permissible limits, presenting substantially elevated health hazards compared to produce from unpolluted regions. Conversely, prevailing research efforts have exclusively addressed vegetable produce or finite subsets of heavy metal(loid) pollutants, and few have focused on the health risks associated with dietary intake of multiple toxic metal(loid)s. Hence, research orientation has progressively moved toward controlling metal(loid) pollutant intensities across edible plant biomass [18]. Nevertheless, numerous previous studies only focused on individual vegetable types or limited metal(loid) elements; integrated assessment on multi-metal(loid) dietary health risks under the riparian water–soil–vegetable continuum remains limited, especially lacking targeted research in Southwest China mining watersheds. Most existing works ignore the linkage of metal(loid) migration among water, riparian soil, farmland soil, and crops, restricting the formulation of systematic pollution control strategies.

In view of the above research progress and existing issues, this study aimed to investigate a typical mining-affected watershed in Southwest China. The three core objectives were: (1) to clarify the spatial distribution and concentration characteristics of As, Pb, Cr, Cd, Cu, Zn, and Ni in surface water, riparian soil, farmland soil, and vegetables; (2) to quantify contamination degree and potential ecological risks using multiple pollution and ecological indices; (3) to evaluate human non-carcinogenic and carcinogenic risks via soil contact and vegetable consumption based on the USEPA framework. The novelty of this work lies in establishing a complete water–riparian soil–farmland soil–vegetable systematic assessment framework, revealing metal(loid) migration rules in riparian mining watersheds, and highlighting the health vulnerability of child populations. The findings can provide scientific references for watershed environmental management and resident health protection.

## 2. Materials and Methods

### 2.1. Sampling Locations and Sample Collection

This study targeted a watershed in Southwest China. Due to confidentiality agreements with local environmental protection bureaus and resident privacy protection, the specific watershed name and precise GPS coordinates cannot be provided. The study area features complex mountain landforms, with the highest altitude reaching 4344.10 m and the lowest altitude of 695 m at the river confluence. It belongs to the subtropical monsoon climate (Köppen Cwa), with an annual average temperature of 20 °C and annual precipitation of approximately 1000 mm. Based on field investigation, land-use type, and mining distribution, the watershed was divided into three functional zones. Zone A: Dominated by continuous agricultural land without upstream mining activities, representing natural background status; Zone B: Riparian farmland with upstream abandoned and active mines, directly affected by mining wastewater and tailing leachate; Zone C: Confluence of small watershed into Jinsha River, with severe river scouring, most farmland destroyed, and only scattered cultivated land remaining. The sampling quantity difference among zones was determined by actual land area, farmland distribution, and accessibility. Zone A has the largest continuous agricultural area and requires more sampling points to reflect background values; Zone B is small in spatial range but concentrated in mining impact; Zone C suffers serious river erosion with extremely limited available sampling sites, and hence only one point was arranged. Sampling was conducted in October 2021. A total of 12 paired surface water and riparian soil samples were collected (6 in Zone A, 5 in Zone B, and 1 in Zone C). Meanwhile, 14 pairs of farmland soil and corresponding vegetable samples were collected.

Surface water was collected at 0.5 m depth using a stratified sampler; three replicates were mixed, stored in polyethylene bottles, transported at 4 °C. Riparian and farmland soils were sampled at 0–20 cm topsoil. Each composite sample was mixed and processed by a coning and quartering method: the mixed soil was piled into a cone, flattened, divided into four quadrants, and two opposite parts were discarded repeatedly until a 500 g representative sample was obtained. Soil samples were air-dried, with coarse organic debris (plant roots, stems, and litter) and rock fragments manually removed, then ground and sieved through 10/60/100-mesh nylon sieves. Vegetables were classified into leafy vegetables, root and tuber vegetables, and fruit vegetables; only edible parts were sampled. Samples were washed with tap water followed by deionized water, inactivated at 105 °C, homogenized, and stored at −20 °C. All vegetable data were calculated based on fresh weight. Refer to Supplementary Table S1 for detailed data.

### 2.2. Analytical Methods for Samples

Field-portable HACH (SL1000, Hach Company, Loveland, CO, USA) equipment was used to synchronously determine the pH and electrical conductivity (EC) of surface water. Soil pH was measured by potentiometry with a water–soil ratio of 2.5:1; soil organic matter content was determined by potassium dichromate titration; and soil particle size composition was analyzed by Malvern laser diffraction method. Water samples were filtered through 0.45 μm membrane filters before detection. Soil and vegetable samples were digested according to USEPA Method 3050B, with superior-grade concentrated nitric acid (Merck KGaA, Darmstadt, Germany) and 30% hydrogen peroxide (Sinopharm Chemical Reagent Co., Ltd., Shanghai, China) as digestive agents. The digestion system included a 0.1 g dry soil sample and 2 g wet vegetable sample. After digestion, 5% (*v*/*v*) dilute nitric acid solution was used to achieve a constant volume of 25 mL, and the solution was filtered through a 0.22 μm membrane for standby. The concentrations of seven heavy metal(loid)s (As, Pb, Cr, Cd, Cu, Zn, and Ni) in all samples were determined by inductively coupled plasma–mass spectrometry (ICP-MS, iCAPQR, Thermo Fisher Scientific, Bremen, Germany). For detailed procedures, see the Supplementary Materials.

### 2.3. Evaluation Indices

#### 2.3.1. Assessment of Heavy Metal(loid) Pollution in Water Bodies

Water environmental quality assessment relies on the comprehensive pollution index as a vital analytical framework, wherein diverse heavy metal(loid)s present across the surveyed region are treated collectively as one unified system, accounting for their mutual interactions and combined ecological impacts on aquatic ecosystems. This method is used to determine the degree and category of heavy metal(loid) pollution in water bodies and to reflect the overall pollution status of the water. The categorization standards for the water quality index (*WQI*) together with pollution severity descriptions appear in Supplementary Table S2. For detailed descriptions, see the Supplementary Materials [19].

#### 2.3.2. Assessment of Heavy Metal(loid) Pollution and Environmental Risk in Soils

For riparian soil and farmland soil, multiple methods were employed to comprehensively evaluate the degree of heavy metal(loid) pollution, and the potential ecological risk associated with multi-heavy metal(loid) contamination in soils was further assessed. First, the pollution index (*PI*) was used to characterize the extent of soil contamination by a single heavy metal(loid)s. In most cases, however, polluted areas are simultaneously affected by multiple heavy metal(loid)s; in such situations, the Nemerow integrated pollution index (*PI_N_*) is applied to represent the comprehensive level of heavy metal(loid) pollution. For detailed descriptions, see the Supplementary Materials [20].

#### 2.3.3. Enrichment Capacity and Pollution Assessment of Heavy Metal(loid)s in the Soil–Vegetable System

Analytical methodologies were implemented to assess heavy metal(loid) pollution in agricultural vegetables from watershed farmland and mining-proximate regions, first establishing vegetables enrichment potential for heavy metal(loid)s, then employing assessment formulas for gauging contamination severity under combined heavy metal(loid) pollution scenarios. As a preliminary step, the bioconcentration factor (*BCF*) served to assess how effectively plant tissues concentrate metal(loid) contaminants. For detailed descriptions, see the Supplementary Materials.

#### 2.3.4. Human Health Risk Assessment

The USEPA health risk assessment model was used to evaluate residents’ non-carcinogenic risk (hazard index, *HI*) and incremental lifetime carcinogenic risk (*ILCR*) via three exposure pathways: soil hand-to-mouth ingestion, dermal contact and inhalation. *HI* < 1 indicates acceptable non-carcinogenic risk. If *ILCR* ≤ 1 × 10^−6^, the carcinogenic risk is considered low; if *ILCR* is between 1 × 10^−5^ and 1 × 10^−4^, it is considered a moderate carcinogenic risk; and if *ILCR* is between 1 × 10^−3^ and 1 × 10^−1^, it is considered a high carcinogenic risk. The parameters needed for calculating the aforementioned equations appear in Supplementary Tables S5 and S6 [21,22].

### 2.4. Quality Control and Statistical Analysis

Strict experimental quality control measures were implemented throughout the whole experimental process to ensure data accuracy and reliability. Blank samples, standard reference materials (GBW10048) and triple parallel samples were set for each batch of sample detection to eliminate system errors and random errors. The standard curve correlation coefficient of all detected heavy metal(loid) elements was greater than 0.999. Descriptive statistics (based on mean value, standard deviation, and coefficient of variation) were used to summarize the data, and all datasets were analyzed with Microsoft Excel (2019 version). Graphical representations, including statistical analyses, were created with Origin 2021 (OriginLab Corp., Northampton, MA, USA).

## 3. Results

### 3.1. Current Status of Heavy Metal(loid) Pollution in Surface Water and Riparian Soils

**Figure 1:**
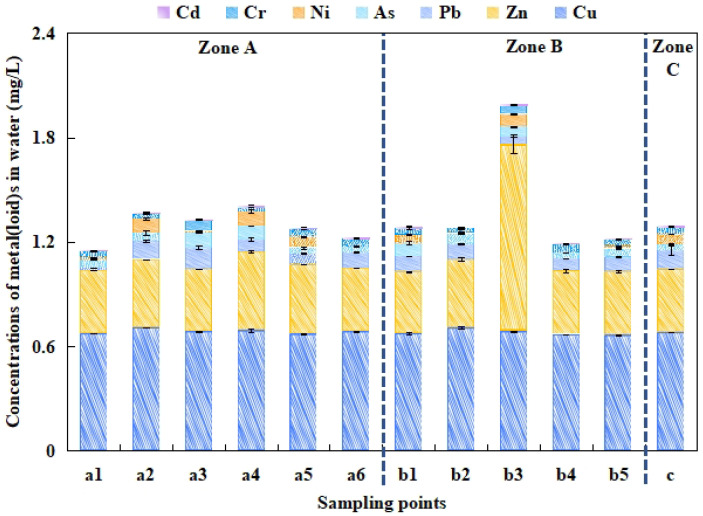
Distribution characteristics of heavy metal(loid)s in water (*X*-axis: a1–a6 for Zone A sampling points; b1–b5 for Zone B sampling points; c for Zone C sampling points).

**Figure 2:**
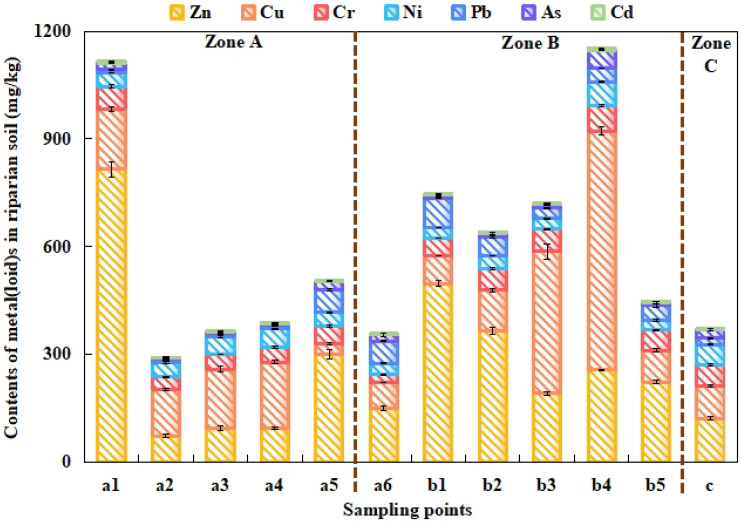
Distribution characteristics of heavy metal(loid)s in riparian soil (*X*-axis: a1–a6 for Zone A sampling points; b1–b5 for Zone B sampling points; c for Zone C sampling points).

The concentrations of seven heavy metal(loid)s in the watershed surface water followed the descending order of Cu > Zn > Pb > As > Cr > Ni > Cd (Figure 1), with 44.0% of samples exceeding the regulatory limits and high-concentration sites predominantly clustered in Zone B (the area with intensive mining activities). The mean concentrations of As, Cd, Pb and Ni exceeded the water quality criteria specified by the WHO and USEPA, whereas those of Cr, Cu and Zn were within the compliant range. In riparian soils, mean concentrations of all seven metal(loid)s remained below China’s agricultural land screening values (GB 15618-2018) (Figure 2) [23]. Yet peak measurements for As, Cd, and Cu, alongside Zn, surpassed their individual limits by factors of 1.98, 4.62, and 1.81, in addition to 2.72, respectively. In relation to regional reference levels from Yunnan/Kunming, average plus peak concentrations for As, Pb, Cd, and Cu, along with Zn all surpassed their corresponding baseline data, while Pb and Cr, together with Ni, exhibited lower average concentrations relative to baseline levels; nevertheless, their peak concentrations uniformly exceeded Yunnan/Kunming regional benchmarks (Supplementary Table S8). These results indicate that while mean soil metal(loid) levels comply with regulatory standards, localized enrichment—particularly for Cd and Cu—may pose risks to adjacent aquatic systems.

### 3.2. Current Status of Heavy Metal(loid) Pollution in the Farmland–Vegetable System

**Figure 3:**
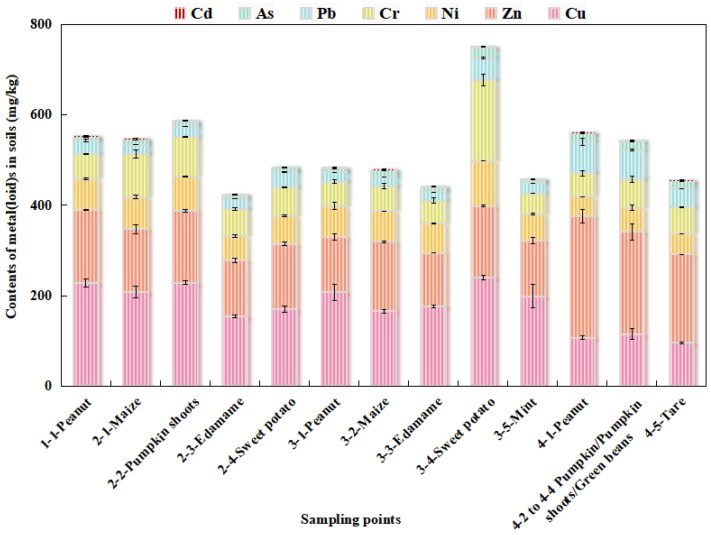
Distribution characteristics of heavy metal(loid)s in farmland soils.

The enrichment degrees of the investigated heavy metal(loid)s (As, Pb, Cr, Cd, Cu, Zn and Ni) in farmland soils differ, with Cu and Zn exhibiting notably higher contents in this area (Figure 3). The concentrations of Cu and Zn range from 96.1 to 214 mg/kg (mean: 177 mg/kg) and from 118 to 268 mg/kg (mean: 161 mg/kg), respectively. The mean concentrations of Pb, Cd and Ni were 0.78–8.83 times the background values of Yunnan and Kunming; As and Cr mean contents exceeded the Yunnan baseline but were lower than the Kunming regional reference values. All sampling sites for Cu in farmland soils exceeded the limit of 100 mg/kg specified in GB 15618-2018, while individual sites for As, Cd and Zn were close to their respective standard thresholds [23].

**Figure 4:**
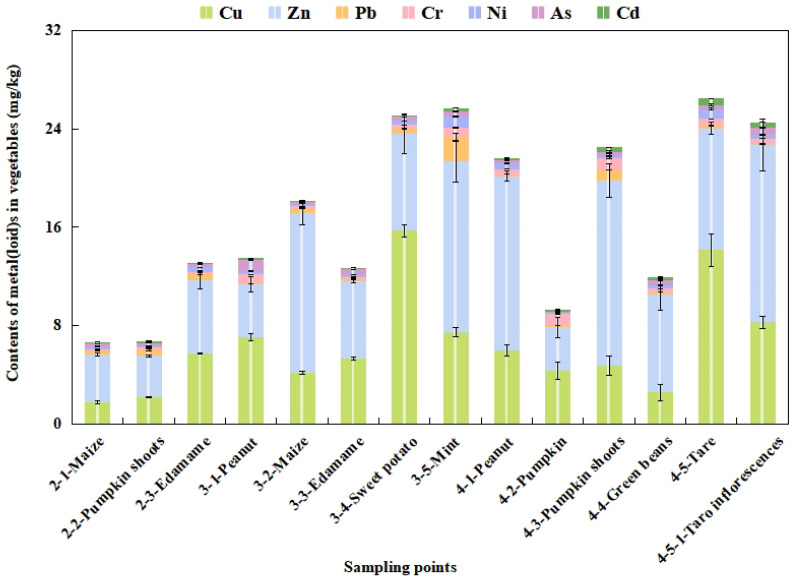
Distribution characteristics of heavy metal(loid)s in vegetables.

The concentrations of heavy metal(loid)s in vegetables grown in farmlands adjacent to the watershed are shown in Figure 4. The ranges (mean values) of As, Pb, Cr, Cd, Cu, Zn and Ni in vegetables are 0.06–0.92 (0.35), 0.07–1.95 (0.41), 0.12–0.99 (0.45), 0.01–0.52 (0.17), 1.76–15.7 (6.39), 3.37–15.0 (8.83) and 0.04–0.93 (0.36) mg/kg, respectively. Among them, the mean values of As and Cr do not exceed the limit of 0.5 mg/kg for fresh vegetables stipulated in the GB 2762-2022, China, whereas the mean values of Pb and Cd exceed the corresponding limits of 0.10 and 0.05 mg/kg, and their maximum values also exceed the limits, indicating that certain vegetables pose a potential consumption risk [24]. When the vegetables are classified into leafy vegetables (pumpkin shoots, mint, and taro inflorescences), root and tuber vegetables (peanut, sweet potato, and taro) and fruit vegetables (maize, edamame, pumpkin, and green beans), Cr, Cu and Zn contents are higher in root and tuber vegetables, whereas Cu, Zn and Pb contents are higher in leafy vegetables. The aggregate accumulation pattern for the seven metal(loid) contaminants revealed the following sequence: root and tuber vegetables > leafy vegetables > fruit vegetables.

**Figure 5:**
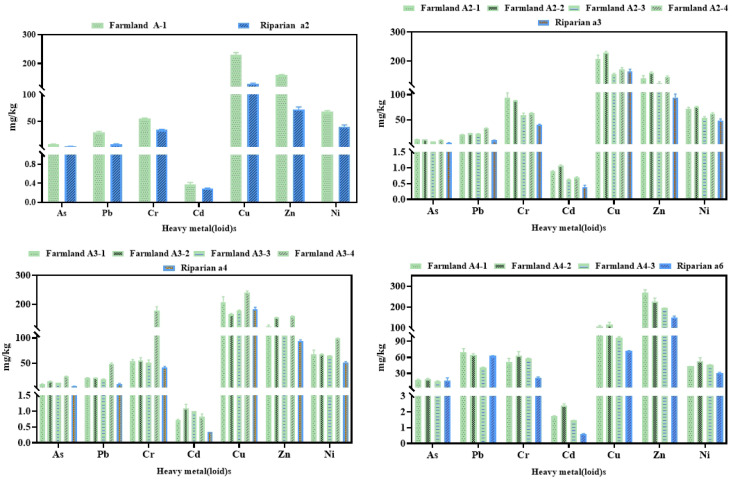
Effects of heavy metal(loid)s in farmland soil on riparian soil loss.

Overall watershed-scale assessment of metal contamination in agricultural–riparian soil continua has been presented above, while spatial interactions whereby vegetablesland metal accumulation affects riparian sediments are depicted in Figure 5. The mean concentrations of the seven heavy metals at farmland sampling sites 1, 2, 3 and 4 are 78.7, 72.8, 76.9 and 74.2 mg/kg, respectively, while those at riparian soils a2, a3, a4 and a6 are 41.3, 51.8, 55.0 and 50.7 mg/kg, respectively. Farmland located upslope exerts a certain influence on the riparian soils. In Area A, field investigation showed that both sides of the watershed are occupied only by farmland; thus, Area A is referred to as the region distant from the mining area. The high heavy metal contents in riparian soils indicate that one of their sources is the farmland on both sides. In addition, the soil particle size is dominated by sand, suggesting that heavy metals in farmland soils can be transported with surface runoff to riparian soils, thereby posing further environmental risks to the aquatic environment.

### 3.3. Environmental Risk Assessment of Heavy Metal(loid)s in the Water–Bankside Soil–Farmland Soil–Vegetable System

**Figure 6:**
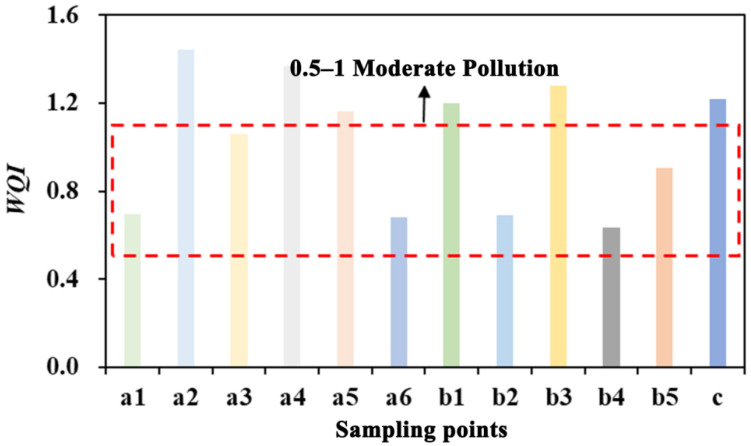
The pollution indexes of heavy metal(loid)s in water (*WQI*).

In this study, the Comprehensive Pollution Index of Water Quality was applied to evaluate the water quality in terms of heavy metal concentrations in the watershed, and the risk was first assessed using the single-factor pollution index for heavy metals in water (*P_water_*), as shown in Table 1. It can be seen that the ranges of As-*P_water_* (0.61–1.69, with an average of 1.06), Pb-*P_water_* (0.28–2.42, with an average of 1.58), and Ni-*P_water_* (0.00–4.08, with an average of 1.87) all fall within the Low Pollution category; the ranges of Cr-*P_water_* (0.62–1.22, with an average of 0.80), Cd-*P_water_* (0.00–1.30, with an average of 0.77), Cu-*P_water_* (0.67–0.71, with an average of 0.68), and Zn-*P_water_* (0.35–1.08, with an average of 0.44) are all classified as No Pollution. Among the seven analyzed metallic pollutants, contamination levels declined following this hierarchy: Ni > Pb > As > Cr > Cd > Cu > Zn, and collectively the aquatic environment displayed minor pollution characteristics. Drawing from mono-factor contamination metrics, an integrated Water Quality Assessment Index for seven metals throughout this drainage basin received evaluation, displayed in Figure 6. The assessment results of the Water Quality Index (*WQI*) for the seven heavy metals showed an average value of 1.03, with a maximum of 1.44 at sampling point a2 in zone A and a minimum of 0.64 at sampling point b4 in zone B. Observations confirm moderate levels of metallic pollutant exposure affecting watershed surface waters, thereby establishing conditions of potential jeopardy for aquatic habitats.

**Figure 7:**
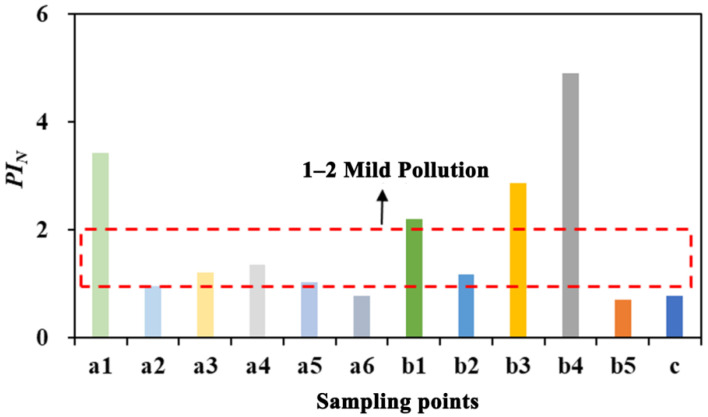
The Nemerow synthetic pollution index (*PI_N_*) of heavy metal(loid)s in riparian soils.

**Figure 8:**
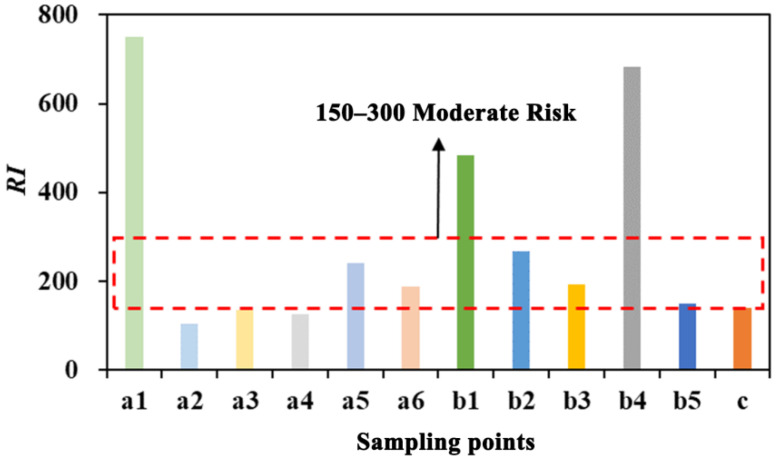
The risk indices (*RI*) of heavy metal(loid)s in riparian soils.

For riparian soils, the single-factor pollution index (*PI*) identified Cd and Cu as low-risk pollution, with the remaining metal(loid)s classified as non-pollution (Table 2), and the pollution intensity following Cu > Cd > Zn > As > Ni > Pb > Cr. The Nemerow integrated pollution index (*PI_N_*) had a mean value of 1.78 (Figure 7), indicating mild combined heavy metal(loid) pollution across the riparian soils; the maximum *PI_N_* of 4.91 at sampling point b4 (Zone B) even reached the heavy pollution level. The Hakanson single-factor ecological risk index (*Ei*) revealed that Cd posed a high ecological risk (mean: 233), while other metal(loid)s were low risk (Table 3), with the ecological threat sequence being Cd > Cu > As > Ni > Pb > Zn > Cr. The comprehensive potential ecological risk index (*RI*) had a mean of 289 (Figure 8), representing moderate ecological risk, and the maximum *RI* of 751 at sampling point a1 (Zone A) reached the high-risk level. For farmland soils, the *PI* ranking was Cu > Cd > Zn > As > Ni > Cr > Pb, with a mean *PI_N_* of 1.36 (mild pollution); the *Ei* followed Cd > Cu > As > Ni > Pb > Cr > Zn, where the maximum *Ei* of Cd reached 603 (extremely high risk), and the mean *RI* of 319 indicated a considerable ecological risk (Table 4). The bioconcentration factors (*BCFs*) of heavy metal(loid)s in vegetables (Table 5) varied by vegetable type: leafy vegetables followed Cd > Zn > Pb > As > Cu > Cr > Ni; fruit vegetables Cd > Zn > Cu > As > Pb > Cr > Ni; and root and tuber vegetables Cd > Cu > Zn > As > Cr > Ni > Pb. The *PI* assessment of vegetables (Table 6) showed that leafy vegetables were heavily polluted (*PI_N_* = 6.34) with the pollution order Pb > Cd > Ni > Cr > As > Zn > Cu; fruit vegetables were mildly polluted (*PI_N_* = 1.92) with the same *PI* sequence; and root and tuber vegetables were moderately polluted (*PI_N_* = 2.90) following Cd > Pb > Ni > Cu > Cr > As > Zn.

### 3.4. Health Risk of Heavy Metal(loid) Exposure in Surrounding Residents

To evaluate health threats from soil-borne heavy metal(loid) pollutants to local populations, we applied an assessment framework proposed by the U.S. Environmental Protection Agency (EPA). The non-carcinogenic risk exposure doses of heavy metal(loid)s in soil through hand-to-mouth exposure are shown in Table 7. Compared to adults, children are at a higher probability of facing non-carcinogenic risks when exposed to soil contaminants via the hand-to-mouth pathway. Long-term exposure to heavy metal(loid)s not only causes non-carcinogenic diseases, but it can also lead to cancer. This study primarily considered the cancer risk faced by residents due to exposure to five heavy metal(loid)s: As, Pb, Cr, Cd, and Ni. The carcinogenic risk (*CR*) value exceeding 1 × 10^−6^ indicates a potential cancer risk. The carcinogenic risk of heavy metal(loid)s in soil through hand-to-mouth exposure is shown in Table 8. Among the adult cohort, cancer risk assessments parallel the juvenile findings, where As, Cr, and Ni *CR* values beyond 1 × 10^−6^ reflect moderate oncogenic hazards. Hand-to-mouth ingestion of soil-borne contaminants poses disproportionately higher oncogenic threat levels for pediatric cohorts when contrasted with mature populations. Dietary exposure assessment revealed that non-carcinogenic risks from vegetable consumption were below the safe level (*HI* < 1) for both children and adults. However, carcinogenic risks from dietary intake were notable: for children, *ILCR* values ranged from 3.16 × 10^−6^ to 5.43 × 10^−6^, approaching the moderate risk category. Ni and As were the primary contributors to dietary cancer risk. These findings suggest that while immediate non-carcinogenic effects from vegetable consumption are low, long-term exposure to As and Ni through diet may pose health concerns, particularly for children.

Dose levels related to non-oncogenic impacts resulting from metal(loid) element consumption through different agricultural products appear in Table 9. For children, As was the main contributor in vegetables. Among mature individuals, As dominated the *HQ* metric contributions from distinct metal elements via ingestion pathways within three designated areas, and non-tumor-related threat indices stayed beneath permissible boundaries (*HI* < 1), implying that consumption-based non-cancerous hazards demonstrate minimal health implications for community inhabitants. Cancer-related threat levels from metal(loid) element contamination among diverse plant-based food varieties stemming from ingestion routes in separate regions appear in Table 10. Among pediatric populations, As and Ni demonstrated elevated cancer risk indices through food consumption pathways compared with remaining metallic contaminants, with Ni surpassing the 1 × 10^−6^ threshold, thereby suggesting possible oncogenic hazards. The *ILCR* values are greater than 1 × 10^−6^ but do not exceed 1 × 10^−5^, suggesting that the carcinogenic risk lies between low and moderate levels. The results for adults consuming different vegetables types are similar to those for children. Overall, children exhibit higher carcinogenic risks via dietary exposure than adults.

## 4. Discussion

### 4.1. Factors Controlling the Environmental Risk of Heavy Metal(loid)s in the Water–Soil–Vegetable System

Aquatic quality evaluation represents an essential component within aqueous resource governance and may be achieved through analyzing the physicochemical characteristics of aquatic systems. When a water body is impacted by external acidic or alkaline pollution, its pH may undergo substantial changes, which can affect microbial activity and reduce the water’s natural purification ability. The World Health Organization (WHO) has specified an acceptable pH range (6.5–8.5) for the health of river and lake waters used as drinking-water sources [25,26]. After various pollutants enter a water body, they undergo environmental processes such as dispersion, accumulation, migration, and dilution under the influence of water flow and natural sedimentation [27]. Consequently, the spatial distribution of pollutants within a watershed exhibits heterogeneity [28].

The riparian zone can act as an important sink for various pollutants, including heavy metal(loid)s, and the retention and accumulation of heavy metal(loid)s in its soils, as well as their spatial variability, may lead to their transfer to neighboring aquatic systems via various hydrological pathways including overland flow, percolation, and sediment mobilization [29,30]. Various physicochemical parameters, particularly soil pH and organic matter (OM) content, exert dominant control over heavy metal(loid) behavior across riparian environments, including their bioaccessibility, toxicity, mobility, and migration potential [31,32]. Bankside soils show considerable heterogeneity in their organic content, requiring regional authorities to prioritize this aspect for elevating riparian substrate quality basin-wide and maintaining clean water conditions.

For enhanced characterization of heavy metal(loid) abundance within the watershed’s surface water, mean values for the seven target metal(loid)s were evaluated relative to findings from other Chinese and global river systems. The findings revealed that the majority of metal(loid) levels across watershed water exceeded those documented in other rivers (Supplementary Table S7), reflecting substantial variability in heavy metal(loid) loadings among diverse river systems [19,33,34,35,36,37,38,39,40,41]. These differences are attributed to the transport dynamics governing metal(loid) behavior within water, which are controlled by physical, hydrological, environmental, and biological processes, as well as chemical reactions with other system components (e.g., advection, dispersion, adsorption/desorption, sedimentation, resuspension, and settling) [42,43,44].

The coefficients of variation (*CVs*) for As, Pb, Cd, Cu, and Zn were >36%, ranging from 73.1% to 98.7%, indicating high variability, whereas the *CVs* of Cr and Ni ranged between 15% and 36%, indicating moderate variability, at 27.2% and 29.29%, respectively (Supplementary Table S8). High *CV* metrics reflect pronounced variability characterizing As, Pb, Cd, Cu, and Zn distribution, which can be ascribed to surrounding cultivated land impacts and additional anthropogenic disturbances. Analogous to surface water metal(loid) contamination profiles, riparian soil samples displayed pronounced metal(loid) enrichment primarily within Zone B (the extractive industry sector). The relatively high metal(loid) content at Sampling Site a-1 in Zone A is likely related to the presence of farmland on both sides of the river, where deployment of selected agricultural agents for soil enrichment and pest suppression purposes has partially intensified concentrations of metal(loid) pollutants. For the present research, we benchmarked metal(loid) content within watershed riparian soils against data reported by studies conducted across China and additional Asian nations (Supplementary Table S8) [23,30,45,46,47,48,49,50]. The comparison revealed that, due to differences in sampling locations, in conjunction with spatial variations in economic maturity levels and progressive environmental safeguarding practices, metal(loid) deposition and accumulation occur across stream bed materials, exhibiting persistence throughout extensive temporal scales.

Agricultural landscapes characterize the riparian zones of the examined watershed, where anthropogenic interventions—specifically through the employment of chemical farming inputs—have influenced metal(loid) build-up in proximal edaphic environments, as these substances inherently contain diverse metal(loid) components, namely As, Pb, Cr, Cd, Cu, Zn, and Ni [51]. The sources of Pb are more strongly associated with transportation, including vehicle exhaust and tire wear, as the entire watershed is located in a mountain valley with a highway running through it; conversely, agricultural practices, particularly the deployment of certain fungicidal agents and soil conditioners, also influence As, Pb and Cu levels [52]. In addition, numerous studies have indicated that Cr is largely derived from parent materials, and it may also enter aquatic systems via multiple forms of wastewater along with agricultural nutrient inputs [53]. The elevated Ni concentration may also be related to the local geological and geomorphological conditions [54]. Overall, heavy metal(loid) contamination in the water mainly originates from agricultural wastewater and domestic sewage, followed by combined factors such as atmospheric deposition; for example, Pb, Zn, Cd and Cu are commonly derived from mineral mining and smelting [54,55].

In this study, farmland soils were subjected to varying degrees of heavy metal(loid) contamination. The overall *PI_N_* values indicate that farmland soils are in a state of mild pollution, mainly by Cu, Cd and Zn, which is likely related to agricultural activities such as fertilizer application [31]. Cd and As are accessory elements in metal(loid) ores and can be released during smelting processes, while agricultural practices such as the application of phosphate fertilizers and pesticides may also promote the accumulation of Cd and As in farmland soils [56]. The study evaluated metal(loid) pollution levels in agricultural produce from farmland alongside vegetables bioaccumulation potential for soil-borne heavy metal(loid)s, and it was found that vegetables exhibited a relatively strong enrichment capacity for Cr, Ni, Cu and Pb. Comparisons among different vegetables types further revealed that leafy vegetables and fruit vegetables had higher enrichment capacities. In addition, direct foliar uptake is also considered a major pathway for atmospheric heavy metal(loid)s, as these pollutants can be absorbed through leaf stomata [57].

### 4.2. Pollution Intensity and Ecological Risk of Heavy Metal(loid)s at the Water–Land–Biota Interface

Application of the comprehensive pollution index of water quality for evaluating aquatic conditions based on metal(loid) content across watershed aquatic systems demonstrated overall low contamination levels. Throughout this work, Ni registered the peak *P_water_* value across surface aquatic samples, corroborating data published by Yan et al. for the Yunnan section of the Yangtze River watershed, and the elevated Ni concentration may also be related to the local geological and geomorphological conditions [54]. However, the water quality index (*WQI)* based on seven heavy metal(loid)s shows that the watershed surface water is currently subject to moderate heavy metal(loid) pollution and that the aquatic environment is in a state of risk.

Riparian soil is also one of the important components of the aquatic environment. Based on *PI_N_* analysis, riparian earth materials within the studied watershed exhibit mild contamination attributable to multi-element heavy metal(loid) loading, implying a potential risk to the riparian soil–water environmental interface. The predominant agricultural land use characterizing both watershed banks in this locality substantially drives heavy metal(loid) deposition patterns observed in adjacent riparian substrates. Multiple pathways introduce heavy metal(loid)s, encompassing vegetable cultivation, household effluent disposal, and vehicular transport; moreover, agricultural anthropogenic practices—particularly synthetic fertilizer use, pesticide implementation, and sewage irrigation—facilitate additional metal(loid) loading in riparian substrates [30,58].

Cd and As demonstrate toxicity profiles exceeding alternative metal(loid) species, leading to elevated ecological hazard potential. The riparian zone can act as both a sink and a source of heavy metal(loid)s. While it retains heavy metal(loid)s from surrounding areas, accumulated metal(loid)s can be remobilized and released back into the aquatic system through processes such as soil erosion, leaching, and sediment resuspension, especially during extreme hydrological events. This bidirectional exchange at the water–land interface creates complex ecological risks for aquatic organisms and terrestrial biota in the riparian zone.

Vegetables, as an important component of the terrestrial biota, can bioaccumulate heavy metal(loid)s from both soil and atmospheric deposition. The relatively strong enrichment capacity of vegetables for Cr, Ni, Cu and Pb, particularly leafy and fruit vegetables, indicates that heavy metal(loid)s can transfer from the abiotic environment to the biotic component of the ecosystem. This bioaccumulation not only affects the growth and development of vegetables themselves but also poses ecological risks to higher trophic levels in the food chain, including humans.

### 4.3. Human Health Risk of Heavy Metal(loid) Exposure in the Watershed

The principal media facilitating human exposure involve soil particles bearing contaminants and polluted food items, both of which carry the capacity to endanger the health status of local population groups. Heavy metal(loid)-containing food intake progressively reduces the physiological reserves of crucial nutrients, whereas prolonged Pb, Cd and As contact may precipitate various pathological outcomes such as neuropathic symptoms, renal functional impairment, hepatic tissue damage, malignancies of pulmonary and cutaneous origin, and osseous fractures [59]. Hence, unprocessed intake of foliar vegetable produce might create serious dangers for community residents’ physiological safety.

In this study, among the three exposure pathways, hand-to-mouth exposure made the greatest contribution to heavy metal(loid) exposure from soil, followed by dermal contact, whereas the contribution of inhalation exposure was almost negligible. Regarding hand-to-mouth exposure pathways, pediatric populations exhibited elevated non-carcinogenic risk levels compared with adult cohorts, primarily attributable to the heightened propensity of younger individuals toward hand-to-oral activities including digit sucking, thereby amplifying dermal-to-oral ingestion hazards [60,61]. When oral intake facilitates heavy metal(loid) incorporation into systemic circulation, they are more easily absorbed, and, coupled with the fact that children are in a stage of rapid growth and development—with some internal metabolic organs being immature and detoxification capacities weaker than those of adults—they are thus more sensitive to the toxic effects of heavy metal(loid)s [62,63,64].

Mature and pediatric populations within the investigated region encounter cancer-related hazards via hand-to-mouth exposure and skin contact pathways, with juvenile subjects experiencing elevated oncogenic risk levels through the hand-to-mouth exposure route compared to grown individuals. Due to distinctive physiological attributes and behavioral patterns, pediatric populations demonstrate heightened susceptibility to environmental heavy metal(loid) contact, a pattern that aligns with observations regarding non-carcinogenic hazard outcomes [65,66]. Overall, when residents come into contact with toxic metal(loid)s, adverse effects on their health may occur, and prolonged exposure to specific carcinogenic metal(loid)s may lead to cancer, with the associated cancer risk increasing over time [67,68].

These factors are subject to considerable uncertainty, and the actual situation may not be as severe as the assessment suggests. Because the toxic effects of heavy metal(loid)s after entering the human body cannot be accurately simulated by health risk assessment models, there exists a necessity to more comprehensively define how pollutant exposure influences health outcomes in human subjects [69,70]. Future research should focus on long-term epidemiological studies to establish more accurate dose–response relationships, as well as on developing more refined health risk assessment models that incorporate factors such as individual susceptibility, combined exposure to multiple pollutants, and different exposure scenarios.

## 5. Conclusions

This study systematically investigated the pollution characteristics, spatial distribution and ecological-health risks of seven heavy metals (As, Pb, Cr, Cd, Cu, Zn, and Ni) in the water–soil–vegetable continuum of a typical mining-affected watershed in Southwest China. The main conclusions are as follows. (1) Mining as the primary driver: Intensified mining activities (Zone B) are the primary source of metal(loid) pollution, leading to moderate contamination of surface water and mild-to-considerable ecological risk in riparian and farmland soils. (2) Food chain risk: Despite mean soil concentrations complying with national standards, local leafy vegetables show severe contamination by lead (Pb) and cadmium (Cd), posing a direct and imminent threat to dietary safety. This highlights the inadequacy of relying solely on soil quality standards for predicting food safety risks. (3) Vulnerable population: Children are at significantly higher non-carcinogenic and carcinogenic risks than adults, primarily through the hand-to-mouth exposure pathway from soil and dietary intake of vegetables contaminated with As and Ni. This study only conducted single-season sampling and did not consider the impact of heavy metal(loid) chemical speciation on bioavailability. The health risk assessment adopted general parameters, which introduces a level of uncertainty. Future research should carry out long-term dynamic monitoring of heavy metal(loid) pollution in the watershed, strengthen the research on heavy metal(loid) speciation and bioavailability, and combine with local residents’ actual exposure behavior and epidemiological investigation to more accurately assess the environmental health risk. At the same time, it is necessary to carry out research on low-accumulation vegetable varieties’ screening and soil heavy metal(loid) passivation technology to ensure the safety of agricultural products in mining areas.

## Figures and Tables

**Table 1:** The single-factor pollution index (*P_water_*) of heavy metal(loid)s in water.

	As	Pb	Cr	Cd	Cu	Zn	Ni
a1	0.93	0.28	0.72	1.30	0.68	0.36	0.60
a2	1.00	2.08	0.62	1.22	0.71	0.39	4.08
a3	1.69	2.42	1.22	0.48	0.69	0.36	0.55
a4	1.59	1.37	0.49	0.88	0.69	0.45	4.09
a5	0.66	1.31	0.89	1.06	0.67	0.40	3.15
a6	0.68	1.80	0.89	0.34	0.69	0.36	0.00
b1	1.56	1.76	0.74	0.92	0.68	0.35	2.39
b2	1.24	1.73	0.57	0.00	0.71	0.39	0.18
b3	1.15	0.83	1.00	0.59	0.69	1.08	3.62
b4	0.61	1.46	0.97	0.37	0.67	0.37	0.00
b5	0.92	1.68	0.79	1.19	0.67	0.37	0.75
c	0.68	2.23	0.74	0.83	0.68	0.36	3.01
*P_water_*	1.06	1.58	0.80	0.77	0.68	0.44	1.87

Note: In this table, italic *P* denotes the single-factor pollution index, and the subscript “water” indicates the matrix evaluated.

**Table 2:** The single-factor pollution index (*PI*) of heavy metal(loid)s in riparian soils.

	As	Pb	Cr	Cd	Cu	Zn	Ni
a1	0.72	0.05	0.25	4.61	1.68	2.72	0.20
a2	0.14	0.04	0.14	0.48	1.30	0.24	0.21
a3	0.19	0.06	0.16	0.64	1.64	0.31	0.26
a4	0.17	0.05	0.17	0.57	1.84	0.31	0.27
a5	0.89	0.38	0.20	1.32	0.30	1.00	0.19
a6	0.68	0.37	0.09	0.98	0.72	0.50	0.17
b1	0.29	0.49	0.20	2.96	0.77	1.66	0.15
b2	0.46	0.30	0.24	1.49	1.12	1.22	0.19
b3	0.39	0.17	0.25	0.76	3.95	0.64	0.15
b4	1.98	0.23	0.28	3.47	6.66	0.85	0.35
b5	0.36	0.24	0.23	0.77	0.87	0.74	0.14
c	0.98	0.10	0.24	0.61	0.89	0.41	0.29
*PI*	0.60	0.21	0.20	1.55	1.81	0.88	0.22

Note: In this table, italic *PI* denotes the single-factor pollution index.

**Table 3:** The potential ecological risk coefficients (*E_i_*) of heavy metal(loid)s in riparian soils.

	As	Pb	Cr	Cd	Cu	Zn	Ni
a1	16.5	1.23	1.83	691	24.7	10.5	5.26
a2	3.29	0.98	1.01	72.5	19.1	0.94	5.59
a3	4.33	1.28	1.20	96.2	24.1	1.21	6.75
a4	3.93	1.20	1.22	85.0	27.0	1.21	7.11
a5	20.3	8.47	1.47	197	4.42	3.85	5.04
a6	15.4	8.25	0.65	146	10.5	1.92	4.34
b1	6.61	10.9	1.45	444	11.4	6.40	4.02
b2	10.4	6.73	1.75	224	16.5	4.71	5.07
b3	8.91	3.87	1.84	114	58.1	2.45	4.02
b4	44.9	5.20	2.05	520	98.0	3.29	9.14
b5	8.22	5.38	1.69	115	12.9	2.86	3.74
c	22.2	2.27	1.76	91.2	13.1	1.57	7.71
*E_i_*	13.7	4.65	1.49	233	26.7	3.41	5.65

Note: In this table, italic *E* denotes the potential ecological risk coefficients, and the subscript “i” indicates the matrix evaluated.

**Table 4:** Pollution assessment of heavy metal(loid)s in farmland soil.

	As	Pb	Cr	Cd	Cu	Zn	Ni	
*PI*								*PI_N_*
Mean value	0.51	0.20	0.28	1.76	1.77	0.54	0.34	1.36
Max. value	0.96	0.41	0.72	4.02	2.41	0.89	0.52	3.01
Min. value	0.28	0.11	0.18	0.63	0.96	0.39	0.23	0.73
*E_i_*								*RI*
Mean value	11.6	4.42	2.08	264	26.0	2.07	8.83	319
Max. value	21.8	9.14	5.26	603	35.4	3.45	13.7	691
Min. value	6.41	2.48	1.36	93.8	14.1	1.52	5.98	126

Note: The italics indicates they follow standard variable/statistical. The single-factor pollution index (*PI*) and the Nemerow synthetic pollution index (*PI_N_*) of heavy metal(loid)s in farmland. The potential ecological risk coefficients (*E_i_*) and the risk indices (*RI*) of heavy metal(loid)s in farmland.

**Table 5:** Bioconcentration factors (*BCFs*) of heavy metal(loid)s in vegetables.

		As	Pb	Cr	Cd	Cu	Zn	Ni
Leafy vegetables	Pumpkin shoots	0.024	0.021	0.002	0.046	0.010	0.021	0.002
	Mint	0.056	0.088	0.017	0.282	0.037	0.113	0.016
	Pumpkin shoots	0.013	0.013	0.016	0.151	0.041	0.067	0.005
	Mean value	0.031	0.041	0.011	0.160	0.029	0.067	0.007
Fruit vegetables	Maize	0.020	0.013	0.001	0.041	0.008	0.028	0.004
	Edamame	0.035	0.019	0.003	0.042	0.037	0.048	0.008
	Maize	0.009	0.017	0.003	0.015	0.025	0.085	0.003
	Edamame	0.040	0.006	0.004	0.037	0.030	0.053	0.003
	Pumpkin	0.003	0.002	0.015	0.070	0.037	0.016	0.003
	Green beans	0.021	0.003	0.004	0.101	0.022	0.035	0.005
	Mean value	0.021	0.010	0.005	0.051	0.027	0.044	0.004
Root and tuber vegetables	Peanuts	0.109	0.004	0.014	0.070	0.034	0.035	0.004
	Sweet potato	0.013	0.008	0.002	0.041	0.065	0.050	0.003
	Peanuts	0.014	0.001	0.009	0.089	0.056	0.053	0.011
	Taro	0.020	0.006	0.009	0.358	0.147	0.051	0.018
	Mean value	0.039	0.005	0.009	0.140	0.076	0.047	0.009

**Table 6:** The single-factor pollution index (*PI*) and the Nemerow synthetic pollution index (*PI_N_*) of heavy metal(loid)s in vegetables.

*PI*								*PI_N_*
	As	Pb	Cr	Cd	Cu	Zn	Ni	
Leafy vegetables	0.76	8.58	1.16	5.37	0.57	0.58	1.36	6.34
Fruit vegetables	0.51	2.52	0.62	1.76	0.40	0.34	0.83	1.92
Root and tuber vegetables	0.91	2.07	1.04	3.80	1.07	0.45	1.58	2.90

Note: The italics indicates they follow standard variable/statistical.

**Table 7:** The non-carcinogenic risk of heavy metal(loid)s in soil from hand-to-mouth exposure of adults and children.

		As	Pb	Cr	Cd	Cu	Zn	Ni	*HI*
		Children							
Hand-to-mouth exposure	Avg.	0.51	0.12	0.28	0.01	0.05	0.01	0.04	1.03
	Max.	0.96	0.24	0.72	0.03	0.07	0.01	0.06	2.10
	Min.	0.28	0.06	0.19	0.00	0.03	0.00	0.03	0.60
Dermal exposure	Avg.	0.04	0.02	0.40	0.01	0.00	0.00	0.00	0.47
	Max.	0.07	0.04	1.01	0.02	0.01	0.00	0.01	1.15
	Min.	0.02	0.01	0.26	0.00	0.00	0.00	0.00	0.30
		Adults							
Hand-to-mouth exposure	Avg.	0.07	0.02	0.04	0.30	0.01	0.00	0.01	0.44
	Max.	0.14	0.03	0.10	0.41	0.01	0.00	0.01	0.70
	Min.	0.04	0.01	0.03	0.16	0.00	0.00	0.00	0.25
Dermal exposure	Avg.	0.07	0.04	0.79	0.01	0.01	0.00	0.01	0.94
	Max.	0.13	0.09	2.01	0.03	0.01	0.00	0.01	2.29
	Min.	0.04	0.02	0.52	0.01	0.01	0.00	0.01	0.60

Note: The italics indicates they follow standard variable/statistical.

**Table 8:** The carcinogenic risk of heavy metal(loid)s in soil from hand-to-mouth exposure of adults and children.

		As	Pb	Cr	Cd	Ni	*ILCR*
		Children					
Hand-to-mouth exposure	Avg.	1.99 × 10^−05^	2.95 × 10^−07^	3.66 × 10^−05^	4.14 × 10^−07^	1.12 × 10^−04^	2.92 × 10^−04^
	Max.	3.72 × 10^−05^	6.09 × 10^−07^	9.26 × 10^−05^	9.47 × 10^−07^	1.75 × 10^−04^	4.89 × 10^−04^
	Min.	1.09 × 10^−05^	1.65 × 10^−07^	2.39 × 10^−05^	1.47 × 10^−07^	7.61 × 10^−05^	1.88 × 10^−04^
Dermal exposure	Avg.	1.36 × 10^−06^	1.65 × 10^−08^	4.10 × 10^−05^	1.86 × 10^−07^	4.73 × 10^−07^	4.30 × 10^−05^
	Max.	2.54 × 10^−06^	3.41 × 10^−08^	1.04 × 10^−04^	4.26 × 10^−07^	8.87 × 10^−07^	1.08 × 10^−04^
	Min.	7.47 × 10^−07^	9.25 × 10^−09^	2.67 × 10^−05^	6.62 × 10^−08^	2.61 × 10^−07^	2.78 × 10^−05^
Inhalation exposure	Avg.	5.51 × 10^−09^	4.01 × 10^−11^	8.47 × 10^−08^	1.89 × 10^−10^	6.87 × 10^−10^	9.11 × 10^−08^
	Max.	1.03 × 10^−08^	8.30 × 10^−11^	2.14 × 10^−07^	4.33 × 10^−10^	1.29 × 10^−09^	2.27 × 10^−07^
	Min.	3.04 × 10^−09^	2.25 × 10^−11^	5.53 × 10^−08^	6.73 × 10^−11^	3.78 × 10^−10^	5.88 × 10^−08^
		Adults					
Hand-to-mouth exposure	Avg.	1.11 × 10^−05^	1.65 × 10^−07^	2.05 × 10^−05^	2.32 × 10^−07^	1.74 × 10^−04^	2.75 × 10^−04^
	Max.	2.08 × 10^−05^	3.41 × 10^−07^	5.18 × 10^−05^	5.30 × 10^−07^	2.37 × 10^−04^	4.12 × 10^−04^
	Min.	6.13 × 10^−06^	9.25 × 10^−08^	1.34 × 10^−05^	8.25 × 10^−08^	9.45 × 10^−05^	1.57 × 10^−04^
Dermal exposure	Avg.	1.08 × 10^−05^	1.32 × 10^−07^	3.27 × 10^−04^	1.49 × 10^−06^	2.36 × 10^−06^	3.42 × 10^−04^
	Max.	2.03 × 10^−05^	2.72 × 10^−07^	8.27 × 10^−04^	3.40 × 10^−06^	3.68 × 10^−06^	8.55 × 10^−04^
	Min.	5.96 × 10^−06^	7.38 × 10^−08^	2.13 × 10^−04^	5.28 × 10^−07^	1.60 × 10^−06^	2.22 × 10^−04^
Inhalation exposure	Avg.	1.23 × 10^−08^	3.23 × 10^−08^	1.90 × 10^−07^	4.24 × 10^−10^	3.43 × 10^−09^	2.38 × 10^−07^
	Max.	2.31 × 10^−08^	6.68 × 10^−08^	4.80 × 10^−07^	9.69 × 10^−10^	5.34 × 10^−09^	5.76 × 10^−07^
	Min.	6.80 × 10^−09^	1.81 × 10^−08^	1.24 × 10^−07^	1.51 × 10^−10^	2.32 × 10^−09^	1.51 × 10^−07^

Note: The italics indicates they follow standard variable/statistical.

**Table 9:** The non-carcinogenic risk of heavy metal(loid)s in dietary exposure of adults and children.

		As	Pb	Cr	Cd	Cu	Zn	Ni	*HI*
Children	Leafy vegetables	0.02	0.00	0.00	0.00	0.00	0.00	0.00	0.04
	Fruit vegetables	0.02	0.00	0.00	0.00	0.00	0.00	0.00	0.02
	Root and tuber vegetables	0.03	0.00	0.00	0.00	0.00	0.00	0.00	0.04
Adults	Leafy vegetables	0.00	0.00	0.00	0.00	0.00	0.00	0.00	0.01
	Fruit vegetables	0.00	0.00	0.00	0.00	0.00	0.00	0.00	0.00
	Root and tuber vegetables	0.01	0.00	0.00	0.00	0.00	0.00	0.00	0.01

Note: The italics indicates they follow standard variable/statistical.

**Table 10:** The carcinogenic risk of heavy metal(loid)s in dietary exposure of adults and children.

		As	Pb	Cr	Cd	Ni	*ILCR*
Children	Leafy vegetables	8.82 × 10^−07^	1.13 × 10^−08^	4.51 × 10^−07^	1.58 × 10^−07^	3.51 × 10^−06^	5.43 × 10^−06^
	Fruit vegetables	5.89 × 10^−07^	3.33 × 10^−09^	2.39 × 10^−07^	5.20 × 10^−08^	2.47 × 10^−06^	3.16 × 10^−06^
	Root and tuber vegetables	1.06 × 10^−06^	2.73 × 10^−09^	4.04 × 10^−07^	1.12 × 10^−07^	6.65 × 10^−06^	4.21 × 10^−06^
Adults	Leafy vegetables	6.58 × 10^−07^	8.44 × 10^−09^	3.37 × 10^−07^	1.18 × 10^−07^	2.62 × 10^−06^	4.05 × 10^−06^
	Fruit vegetables	4.40 × 10^−07^	2.48 × 10^−09^	1.78 × 10^−07^	3.88 × 10^−08^	1.84 × 10^−06^	2.36 × 10^−06^
	Root and tuber vegetables	7.89 × 10^−07^	2.04 × 10^−09^	3.01 × 10^−07^	8.36 × 10^−08^	4.96 × 10^−06^	3.14 × 10^−06^

Note: The italics indicates they follow standard variable/statistical.

## Data Availability

The original contributions presented in this study are included in the article/Supplementary Materials. Further inquiries can be directed to the corresponding author.

## Supplementary Materials

The following supporting information can be downloaded at: https://www.mdpi.com/article/10.3390/toxics14060539/s1, Table S1: The information list of sampling point. Table S2: The water quality grading standard of *WQI*. Table S3: Classification of soil pollution by heavy metals based on the Nemerow pollution index. Table S4: Classification of potential ecological risk of soil heavy metal pollution based on the risk factor (*Ei*) and risk index (*RI*). Table S5: Parameter definitions and reference values. Table S6: Parameters used in *HQ* and *ILCR.* Table S7: Statistical analysis of date about heavy metal concentrations in water and comparison with others rivers (mg/L). Table S8: Statistical analysis of date about heavy metal concentrations in riparian soils and comparison with others rivers (mg/kg).
