# Supplementary material for: Heavy Metal(loid) Pollution Characteristics and Risk Assessment in the Water–Soil–Vegetable System of a Watershed in Southwest China
Source: Toxics. 2026 Jun 22;14(6):539. doi: 10.3390/toxics14060539 (PMC13308475; doi:10.3390/toxics14060539)
Supplement: Supplementary file 1 [file toxics-14-00539-s001.zip › toxics-4318436-supplementary.pdf]

Heavy Metal(loid) Pollution Characteristics and Risk Assessment in the Water–Soil–Vegetable System of a Watershed in Southwest China

Mengying Li [a], Jinjie Zhao [a], Wenjing Shen [a], Duanyang Yuan [a], Chengchen Wang [b], Ping Xiang [b*]

[a] Yunnan Open University, Kunming 650023, China

[b] Institute of Environmental Remediation and Human Health, School of Ecology and Environment, Southwest Forestry University, Kunming 650224, China

* Corresponding author,

Institute of Environmental Remediation and Human Health, School of Ecology and Environment, Southwest Forestry University, 650224, China

E-mail addresses: xiangping@swfu.edu.cn or ping_xiang@126.com (P. Xiang)

2. Materials and Methods

2.1 Sampling Locations and Sample Collection

In addition, 14 pairs of farmland soil and corresponding vegetables samples were collected. Refer to Supplementary Table S1 for detailed data.

Supplementary Table S1. Information list of sampling points.

Sampling point	Elevation, m	Samples
**a1**	1405.70	Surface water and bankside soil
**a2**	1420.81	
**a3**	1401.91	
**a4**	1396.85	
**a5**	1300.88	
**a6**	1258.76	
**b1**	1065.59	Surface water and bankside soil
**b2**	1056.76	
**b3**	944.63	
**b4**	872.87	
**b5**	811.95	
**c**	748.68	
**A-1**	1420.81	Farmland soil
**A-2**	1401.91	Maize, pumpkin shoots (tender pumpkin shoots), edamame
**A-3**	1396.85	Peanuts, maize, edamame, sweet potato, mint
**A-4**	1182.28	Peanuts, pumpkin, pumpkin shoots, green beans, taro, taro inflorescences

Note: Vegetables and farmland soils were collected as matched pairs.

Surface water in the watershed was collected using a water-quality-stratified sampler. Triplicate surface water samples were pooled in polyethylene bottles and delivered to laboratory facilities the same day, then maintained under 4 °C refrigerated conditions. Per water monitoring location, triplicate riparian soil specimens were obtained from riverbank zones, rigorously mixed to generate a composite sample, and quartered using the quartering method, leaving approximately 500 g, which was placed in a sample bag and properly labeled. Once received at analytical facilities, soil materials underwent atmospheric drying; coarse organic matter (plant roots) coupled

with lithic fragments were discarded, after which samples experienced milling and fractionation via nylon mesh screens at multiple sizes (10, 60, and 100 mesh). The sieved fractions were stored separately at room temperature for subsequent analysis. In the farmland areas, surface soil was collected using wooden tools to avoid contamination by other metals, following an S-shaped (snake-like) sampling pattern. Per individual sampling location, six topsoil subsamples were obtained and rigorously combined to generate a composite sample, which was then quartered using the quartering method, leaving approximately 500 g, placed in a sample bag, and labeled. Once received at analytical facilities, soil materials underwent atmospheric drying; coarse organic matter (plant roots) coupled with lithic fragments were discarded, after which samples experienced milling and fractionation via nylon mesh screens at multiple sizes (10, 60, and 100 mesh). The sieved fractions were stored separately at room temperature for subsequent analysis. The collected vegetables were roughly classified into root and tuber vegetables, leafy vegetables, and fruit vegetables, and only the edible parts were sampled. Vegetable sampling took into account both the local yield and sowing area of different vegetables, as well as the cultivation practices (open-field cultivation and greenhouse cultivation), so that the samples had good representativeness and reflected the daily dietary patterns of local residents. Per sampling site, between three to five plant individuals were gathered, then merged to constitute one mixed sample, enclosed in properly marked polyethylene packaging, ensuring airtight sealing to inhibit moisture depletion throughout specimen conveyance to analytical facilities. The samples were transferred to a low-temperature environment

within 24 h. The edible portions of vegetable samples were first rinsed thoroughly with tap water, followed by 3–5 rinses with deionized water. The samples were then blanched in an oven at 105 °C for 30 min, homogenized using a food processor, stored in clean food-grade bags with clear labels, and preserved in a freezer at –20 °C.

2.2 Analytical methods for samples

Field-based assessment of aquatic physicochemical characteristics (pH and EC—electrical conductivity) was performed using portable HACH analytical equipment. The pH value of soil samples was measured using potentiometry with water and soil proportioned at 2.5:1. Specifically, soil portions (10 g, 10-mesh sieved) were placed in 50 mL volumetric beakers and mixed with 25 mL ultrapure water. Following intensive manual agitation with glass rods (1–2 min duration), the resultant suspension was allowed to equilibrate for 30 min under conditions avoiding atmospheric nitrogen or volatile acidic gas influence, subsequently subjected to pH analysis through calibrated instrumentation. Soil granulometric composition was assessed using Malvern laser diffraction technology, with size fractions categorized following international classification standards. Potassium dichromate titrimetric analysis was employed to determine soil organic matter concentrations.

Prior to analysis, water specimens passed through 0.45 μm membrane filtration units, and the permeates obtained were placed into labeled, coded sample vessels. The concentrations of seven heavy metals, including As, Pb, Cr, and Cd, in the filtrates were then determined by inductively coupled plasma–mass spectrometry (ICP-MS, iCAPQR, Thermo Fisher Scientific Co., Ltd.).

USEPA Method 3050B was employed for digesting soil and plant tissue samples, using concentrated nitric acid and 30% hydrogen peroxide. The digestion was performed on 0.1 g of soil (dry weight) and 2 g of vegetables/mining-area dominant plant samples (wet weight). Post-digestion solutions underwent volumetric adjustment to 25 mL by adding 5% nitric acid (Merck). Prior to ICP-MS analysis of heavy metal concentrations, the solutions were filtered through 0.22 μm aqueous membrane filters. Each treatment was performed in triplicate. To ensure analytical quality, standard reference materials, including a blank standard and a certified reference material (GBW10048, *celery* GSB-26, provided by the Institute of Geophysical and Geochemical Exploration, Chinese Academy of Geological Sciences), were used.

2.3 Evaluation indices

2.3.1 Assessment of heavy metal pollution in water bodies

Water environmental quality assessment relied on the comprehensive pollution index as a vital analytical framework, wherein diverse heavy metals present across the surveyed region are treated collectively as one unified system, accounting for their mutual interactions and combined ecological impacts on aquatic ecosystems. This method is used to determine the degree and category of heavy metal pollution in water bodies and to reflect the overall pollution status of the water. The categorization standards for the water quality index *(WQI)* together with pollution severity descriptions appear in Supplementary Table 2, and the mathematical expression is given by:

$$P_{water}=\frac{C_{water}}{C_{wb}} \tag{1}$$

$$WQI=\frac{1}{n}\sum_{1}^{n} P_{water} \quad (2)$$

The term P_{water} expresses the pollutant index (*PI*) pertaining to heavy metal species dissolved in water; C_{water} corresponds to the observed concentration for heavy metal species in aquatic samples (mg/L); C_{wb} indicates the regulatory standard for heavy metallic elements in water bodies (mg/L), referenced from *Class III of the Environmental Quality Standards for Surface Water* (GB 3838-2002, China; As/Pb/Cr=0.05 mg/L, Cd=0.005 mg/L, Cu/Zn=1 mg/L, Ni=0.02 mg/L); *WQI* denotes the water quality index; and *n* is the total number of heavy metals in the water[19].

Supplementary Table S2. The water quality grading standard of *WQI*.

Grade	Pollution category	*WQI*
I	Clean	≤ 1
II	Relatively Clean	0.1 ~ 0.3
III	Slight Pollution	0.3 ~ 0.5
IV	Moderate Pollution	0.5 ~ 1.0
V	Heavy Pollution	1.5 ~ 5.0
VI	Severe Pollution	> 5.0

2.3.2 Assessment of heavy metal pollution and environmental risk in soils

For riparian soil and farmland soil, multiple methods were employed to comprehensively evaluate the degree of heavy metal pollution, and the potential ecological risk associated with multi-heavy metal contamination in soils was further assessed. First, the pollution index (*PI*) was used to characterize the extent of soil contamination by a single heavy metal. In most cases, however, polluted areas are

simultaneously affected by multiple heavy metals; in such situations, the Nemerow integrated pollution index (PI_N) is applied to represent the comprehensive level of heavy metal pollution. The formulas are as follows:

$$PI=\frac{C_{soil}}{S_{soil}} \tag{3}$$

$$PI_N=\sqrt{\frac{PI_{ave}^2+PI_{max}^2}{2}} \tag{4}$$

where PI denotes the pollutant index characterizing metallic contaminants within soil matrices; C_{soil} denotes the determined concentration levels of heavy metallic elements in soil samples (mg/kg); S_{soil} corresponds to the applicable environmental quality threshold (mg/kg) governing soil heavy metal content, as specified in *Soil Environmental Quality – Risk Control Standard for Soil Contamination of Agricultural Land* (GB 15618-2018,China). Heavy metal pollution intensity in soils is classified through PI values into three grades: when $PI \leq 1$, pollution is mild; when $1 < PI \leq 3$, pollution is moderate; when $PI > 3$, pollution is strong. PI_N represents the Nemerow integrated pollution index for soil heavy metals; PI_{ave} is the average pollutant index of the various heavy metals; and PI_{max} is the maximum PI among these heavy metals. Pollution level divisions for heavy metals in soil based on PI_N values are provided in Supplementary Table S3.

Supplementary Table S3. Classification of soil pollution by heavy metals based

on the Nemerow pollution index.

**Nemerow integrated pollution index**	**Pollution level**
< 0.7	No Pollution
$0.7 \leq PI_N < 1$	Warning Line
$1 \leq PI_N < 2$	Mild Pollution
$2 \leq PI_N < 3$	Moderate Pollution
≥ 3	Heavy Pollution

The limitations of preceding heavy metal contamination assessments in soils—particularly their inability to comprehensively represent environmental and ecological hazards—necessitated applying Hakanson's (1980) potential ecological risk index (*RI*)[20]. This method is an assessment model established by integrating ecology, environmental toxicology, and other related disciplines. Through comprehensive integration of heavy metal levels, speciation, toxic potential, and environmental responsiveness, this index achieves superior accuracy in characterizing ecological threats posed by heavy metal-laden soils within contaminated areas. The mathematical expressions employed are:

$$E_i = T_i \frac{C_i}{B_i} \quad (5)$$

$$RI = \sum E_i \quad (6)$$

where E_i represents the risk factor of the individual heavy metal *i*. T_i signifies the toxic response factor corresponding to heavy metal *i*, where the values applied herein included: As—10; Pb—5; Cr—2; Cd—30; Cu—5; Zn—1; and Ni—5. C_i is the measured concentration of the heavy metal in soil; B_i is the environmental background value of the *i*-th heavy metal, for which the background values of

Yunnan Province (as mentioned above) were adopted. Using the E_i and RI metrics, the stratification of potential ecological threats from soil heavy metals appears in Supplementary Table S4.

Supplementary Table S4. Classification of potential ecological risk of soil heavy metal pollution based on the risk factor (E_i) and risk index (RI).

E_i	RI	Ecological risk level
$E_i < 40$	$RI < 150$	Low
$40 \leq E_i < 80$	$150 \leq RI < 300$	Moderate
$80 \leq E_i < 160$	$300 \leq RI < 600$	Considerable
$160 \leq E_i < 320$	$600 \leq RI < 1200$	High
$320 \geq E_i$	$1200 \geq RI$	Extremely High

2.3.3 Enrichment capacity and pollution assessment of heavy metals in the soil–vegetable system

Analytical methodologies were implemented to assess heavy metal pollution in agricultural vegetables from watershed farmland and mining-proximate regions, first establishing vegetables' enrichment potential for heavy metals, then employing assessment formulas for gauging contamination severity under combined heavy metal pollution scenarios. As a preliminary step, the bioconcentration factor (BCF) served to assess how effectively plant tissues concentrate metallic contaminants. The formula is expressed as follows:

$$BCF=\frac{C_v}{C_s} \tag{7}$$

where BCF is the bioconcentration factor of heavy metals in the edible parts of

vegetables; C_v is the measured concentration of heavy metals in the vegetables (mg/kg); and C_s is the corresponding heavy metal concentration in the soil where the vegetables were grown (mg/kg). When *BCF* values decrease, this demonstrates weaker metallic contaminant uptake and concentration abilities in vegetables.

The pollution assessment of heavy metal contents in vegetables was conducted using the single-factor pollution index and the Nemerow integrated pollution index, with the calculation formulas identical to Equations (3) and (4) in Section 2.3.2. In this context, C_{soil}, the measured concentration of heavy metals in soil (mg/kg), is replaced by C_{veg}, the measured concentration of heavy metals in vegetables (mg/kg); and S_{soil}, the environmental quality standard limit for heavy metals in soil (mg/kg), is replaced by S_{veg}, the maximum allowable limit of heavy metal contaminants in vegetables (mg/kg). The values of S_{veg} were determined with reference to the *National Food Safety Standard – Maximum Levels of Contaminants in Foods* (GB 2762-2022, China)[24].

2.3.4 Human health risk assessment

Two principal considerations govern how metallic pollutants present within ecological systems threaten human well-being. Initially, environmental contamination severity matters, encompassing metal abundance and corresponding poisoning potential; the second is the exposure pathways in the human body, including the behavior and physicochemical properties of the heavy metal species to which humans are exposed. After entering the human body, heavy metals can adversely affect human health through dermal contact, hand-to-mouth exposure, and inhalation exposure (e.g., adhering to dust). Health hazard estimation methodologies developed by USEPA

facilitate determination and anticipation of adverse effects (genotoxic and systemic health risks) stemming from vegetable/edaphic metal burden within designated study zones[21].

The exposure dose via the hand-to-mouth ingestion pathway is calculated as follows:

$$ADD_{ing}=\left(C\times IngR_i\times CF\times EF\times ED\right)/(BW\times AT) \qquad (8)$$

The exposure dose via the inhalation pathway is calculated as follows:

$$ADD_{inh}=\left(C\times IngR_i\times EF\times ED\right)/(PEF\times BW\times AT) \qquad (9)$$

Calculation of skin-mediated uptake quantities follows this formula:

$$ADD_{der}=(C\times SA\times CF\times AF\times ABS\times EF\times ED)/(BW\times AT) \qquad (10)$$

For the coefficient meanings and precise benchmark values used across these mathematical expressions, refer to Supplementary Table S5.

Supplementary Table S5. Parameter definitions and reference values.

Parameter	Definition	Unit	Reference value	
			Children	Adults
C	Heavy metal content in soil/vegetables	mg/kg	This study	
IngR$_{soil}$	Oral ingestion rate of soil	mg/day	200	100
IngR$_{veg}$	Oral ingestion rate of vegetables	mg/day	300	200
IngR$_{air}$	Inhalation rate	m^3/day	7.5	15
CF	Conversion factor	kg/mg	1×10^{-6}	
EF	Exposure frequency	day/year	350	
ED	Exposure duration	years	6	24
BW	Body weight	kg	15.9	56.8
AT	Averaging time	days	For carcinogenic effects: AT = ED × 365<br>For non-carcinogenic effects: AT = 70 × 365	
PEF	Particulate emission factor	m^3/kg	1.36×10^9	
SA	Exposed skin surface area	cm^2	2800	5700
AF	Skin adherence factor	mg/cm^2/day	0.2	0.7

Parameter	Definition	Unit	Reference value	
			Children	Adults
ABS	Dermal absorption factor	/	Carcinogenic elements: 0.01<br>Non-carcinogenic elements: 0.001	

The toxic effects of various metallic contaminants absorbed by humans via multiple exposure routes vary considerably. The hazard index (*HI*) establishes quantitative measures for systemic (non-carcinogenic) dangers arising when humans encounter heavy metal pollution, determined by adding together pathway-specific *HQ* values corresponding to each toxic metal intake route. Defined as the lifetime cancer risk (*ILCR*) from heavy metal exposure, carcinogenic risk quantifies the statistical likelihood of individuals experiencing the onset of a particular malignancy variant throughout their lifetime due to such pollutant contact[22]. The calculation equations are as follows:

$$HQ=\frac{ADD_{ij}}{RfD_{ij}} \quad (11)$$

$$HI=\sum HQ \quad (12)$$

$$CR=ADD_{ij}\times SF_{ij} \quad (13)$$

$$ILCR=\sum CR \quad (14)$$

These equations employ *i* to designate a particular category of heavy metal and *j* to specify a distinct pathway of exposure; *HQ* denotes the risk level (%); *HI* is the hazard index (%); *RfD* is the reference dose (mg/kg/d); and *SF* is the carcinogenic slope factor (mg/kg/d). The acceptable level for non-carcinogenic risk is $HI < 1$. When the carcinogenic risk *CR* exceeds 1×10^{-6}, there is a potential risk of cancer. If $ILCR \leq 1\times10^{-6}$, the carcinogenic risk is considered low; if *ILCR* is between 1×10^{-5} and 1×10^{-4}, it is considered a moderate carcinogenic risk; and if *ILCR* is between 1×10^{-3} and 1×10^{-1}, it

is considered a high carcinogenic risk. The parameters needed for calculating the aforementioned equations appear in Supplementary Table S6.

Supplementary Table S6. Parameters used in *HQ* and *ILCR*.

	RfD_{ing}	RfD_{inh}	RfD_{der}	SF_{ing}	SF_{inh}	SF_{der}
**As**	3.00×10^{-4}	3.01×10^{-4}	1.23×10^{-4}	1.50	15.1	3.66
**Pb**	3.50×10^{-3}	3.52×10^{-3}	5.25×10^{-4}	0.0085	0.042	0.017
**Cr**	3.00×10^{-3}	2.86×10^{-5}	6.00×10^{-5}	0.50	42.0	20.00
**Cd**	1.00×10^{-3}	2.40×10^{-6}	1.00×10^{-5}	0.38	6.30	6.10
**Cu**	4.00×10^{-2}	4.02×10^{-2}	1.20×10^{-2}	0.30	-	-
**Zn**	3.00×10^{-1}	6.00×10^{-2}	3.00×10^{-1}	0.40	-	-
**Ni**	2.00×10^{-2}	2.06×10^{-2}	5.40×10^{-3}	1.70	0.84	0.16

Note: "–" indicates no data.

4. Materials and Methods

The findings revealed that the majority of metal levels across watershed water exceeded those documented in other rivers (Supplementary Table S7), reflecting substantial variability in heavy metal loadings among diverse river systems.

Supplementary Table S7. Statistical analysis of data regarding heavy metal

concentrations in water and comparison with others rivers (mg/L).

	As	Pb	Cr	Cd	Cu	Zn	Ni	Reference
**Avg.**	0.05	0.08	0.04	0.004	0.68	0.44	0.04	This study
**Max.**	0.08	0.12	0.05	0.004	0.71	1.08	0.08	
**Min.**	0.03	0.04	0.02	0.000	0.66	0.35	0.000	
**SD.**	0.02	0.03	0.01	0.002	0.01	0.20	0.03	
**CV. /%**	36.2	37.6	25.3	53.5	2.1	46.5	89.2	
**Haihe River (China)**	0.003	0.001	0.03	<0.001	0.003	0.03	0.02	[33]
**Challawa River (Nigeria)**	-	0.69	-	0.43	-	0.52	-	[34]
**Nakuvadra–Rakiraki River (Fiji)**	-	0.01	0.13	0.003	0.02	0.05	0.05	[35]
**Fen River (China)**	-	19.2	4.24	0.12	0.79	1.81	7.52	[36]
**Omo River (Ethiopia)**	-	0.32	0.39	-	0.17	0.10	0.01	[37]
**Benin River (Nigeria)**	-	0.18	-	0.01	0.02	0.01	0.42	[38]
**Xijiang River (China)**	0.006	0.002	0.01	<0.001	0.003	0.02	0.001	[39]
**Class III Standard of Environmental Quality Standards for Surface Water**	0.05	0.05	0.05	0.005	1	1	0.02	[19]
**Standards for Irrigation Water Quality**	0.05	0.2	0.1	0.01	1	2	0.2	[40]
**WHO**	0.01	0.01	0.05	0.003	2	1	0.02	[41]
**USEPA**	0.01	0.015	0.1	0.005	1.3	1	-	

Note: "–" indicates no available experimental data.

For the present research, we benchmarked metal content within watershed riparian soils against data reported by studies conducted across China and additional Asian nations (Supplementary Table S8).

Supplementary Table S8. Statistical analysis of data regarding heavy metal

concentrations in riparian soils and comparison with others rivers (mg/kg).

	As	Pb	Cr	Cd	Cu	Zn	Ni	Reference
**Avg.**	**15.1**	**35.2**	**50.9**	**0.93**	**181**	**265**	**40.9**	**This study**
**Max.**	**49.4**	**82.8**	**62.6**	**2.77**	**666**	**815**	**66.1**	
**Min.**	**3.62**	**7.41**	**22.1**	**0.29**	**71.6**	**72.6**	**27.1**	
**SD.**	**12.9**	**25.7**	**13.8**	**0.82**	**179**	**214**	**12.2**	
**CV. /%**	**85.2**	**73.1**	**27.2**	**87.8**	**98.7**	**80.8**	**29.9**	
**Liaohe River (China)**	17	17	34	-	25	31	23	[45]
**Shaying River (China)**	-	17.3	55.4	0.22	20.4	63.8	26.9	[30]
**Huangshui River (China)**	4.70	-	223.7	0.40	13.1	62.3	12.0	[46]
**Shiyang River (China)**	9.94	20.1	62.9	0.15	21.3	56.9	28.5	[47]
**Sutlej River (India)**	1.18	36.8	28.9	2.42	26.3	55.9	28.4	[48]
**River Swat (Pakistan)**	-	19.6	38.1	3.18	23.4	57.7	32.5	[49]
**Soil Environmental Quality – Risk Control Standard for Soil Contamination of Agricultural Land**	25	170	250	0.6	100	300	190	[23]
**Background Values of Soil Elements in Kunming City**	14.9	43.3	86.8	0.18	44.6	99.2	46.1	[50]
**Background Values of Soil Elements in Yunnan Province**	11	37.9	68.1	0.12	34	77.7	36.2	

Note: "–" indicates no available experimental data.

References

The reference numbers cited in this supplementary file correspond to those in the main manuscript. All references are listed in the main text.
